# Supplementary material for: Universal screening for SARS-CoV-2 infection among pregnant women at Elmhurst Hospital Center, Queens, New York
Source: PLoS One. 2020 Dec 10;15(12):e0238409. doi: 10.1371/journal.pone.0238409 (PMC7728244; doi:10.1371/journal.pone.0238409)
Supplement: Attachment — Submitted filename: Response to Reviewers 7.27.20.docx [file pone.0238409.s002.docx]

**RESPONSE TO REVIEWERS**

Thank you for all of the comments and edits to the manuscript. Below please find the reviewer comments (in Bold) with our responses following. Of note, Line numbers refer to the manuscript with Track Changes. Also, we have updated a few of the references and associated information given the time that has lapsed since original submission of this manuscript and the interim publication of a number of relevant articles. These additional references did not meaningly alter the manuscript content.

To respond to the Editor’s comments, we are planning to provide a de-identified dataset as a Supporting Information file. We are going through the process of an ethics committee review of the dataset to evaluate the risk that individuals might be identifiable through indirect identifiers. We will provide this at the earlierst possible date.

**Additional Editor Comments (if provided):**

**I strongly suggest including the socio-economic status and if possible the number of persons per households of the studied population in order to evaluate if Hispanic ethnicity and Spanish as a primary language are or not confounder associated factors.** 

Thank you for this comment. We agree that this would be a valuable analysis to add. Unfortunately, there is limited information on socioeconomic status available to us through the Electronic Medical Record and what is available is largely incomplete. Specifically, information on household income, employment status, household members, and occupation was missing for most of the patients in our sample. We have included insurance status, as that data is available and complete. We now emphasize as a limitation that the cross-sectional association we observed between test-positivity and Hispanic ethnicity and Spanish as a primary language may be confounded by patients' socioeconomic status.

**Reviewer #1: Comments to the Author:**

**The study by Dr. Maru et al. describes the prevalence of SARS-COV-2 infection among pregnant patients that were admitted to a L&D room, at the epicenter of the COVID-19 pandemic in USA. The authors add very valuable information regarding the spectrum of COVID-19 disease in this population; they also address demographic characteristics to their findings.**

**I have some suggestions and questions:

1. Line 91: past tense. “The primary objective of this study was to describe”.**

Thank you, this change was made.

**2. Line 111: In Methods: could you describe were the swabs were taken (nasopharyngeal, oropharyngeal, etc)?**

We performed the SARS-CoV-2 testing from nasopharyngeal swabs. This information is now included in the methods section as requested (line 146—line numbers refer to manuscript with Track Changes).

**3. Line 116: Comorbidities: could you describe which comorbidities were assessed?**

The comorbidities we assessed were as follows: hypertensive disorders (chronic hypertension, gestational hypertension, preeclampsia, superimposed preeclampsia), pre-pregnancy maternal obesity, asthma or pulmonary disease, diabetes (pregestational and gestational), infectious disease (HIV, hepatitis B), depression or anxiety, and other significant maternal disease including heart, kidney, or thyroid disease. We have now added this information to the manuscript (line 156).

**4. Line 133: Table 1. Title: Were all the patients presenting for delivery? You report 12 still pregnant patients. Table 1 per se: Comorbidities (No.) means the number of comorbidities, then you report 1+, this means all the patients had 2 or more comorbidities or had at least one? Could you be more clear? If you wish to express it as yes / none, in the results text you may describe the comorbidities and the presence of more than 1, and may be useful in this scenario to eliminate the Word (No.) in the table.**

The cohort includes all patients admitted to the L&D unit during the study period, thus antepartum admissions were also included in the analysis even though they were not presenting for delivery. The title of Table 1 has been changed to better reflect this.

We wish to express the comorbidities as either none or at least 1 comorbidity. We have deleted the Word (No.) in the table and clarified in the results text to better reflect our intentions regarding reporting the comorbidities.

**5. Line 140: Results: Concerning the symptomatic patients, could you be more thorough about the symptoms? Was the presence of symptoms related to the mode of delivery/preterm delivery? Any fatalities?**

Of those women who were symptomatic, the median number of symptoms on admission was 2 with the most common being fever and cough. Other symptoms that were reported by patients in this cohort included body aches, sore throat, shortness of breath or difficulty breathing, headache, and runny nose.

The presence of symptoms did not appear to be related to the mode of delivery or associated with preterm delivery (see data below). During our study period there were no maternal deaths.

Asymptomatic	Symptomatic

*Mode of delivery*

Vaginal	20	5

Cesarean	10	3

Missing (still pregnant)	3	5

*Time of delivery*

Term	28	7

Pre-term	2	1

Missing (still pregnant)	3	5

We now briefly describe this in the manuscript (line 232).

**6. Line 148: Results: I don´t understand the variable primary language (Spanish) and its relationship with positivity testing: does this mean these patients were not born in USA or are first degree descendants of latin american immigrants, and this relates these characteristics with positivity? Is there a way you could you be more elaborate?**

At Elmhurst Hospital we serve a largely immigrant (foreign-born), resource-poor population. The primary language of Spanish likely indicates the patient was not born in the USA or is a first-degree descendant of Latin American immigrants (although there may be exceptions). Both of these characteristics are common in our patient population. Additionally, Spanish as a preferred language may indicate barriers to seeking or utilizing healthcare.

We have added this information to the discussion section of the paper (line 324).

**7. Lines 149-151: Results: could you add the ORs to the description?**

Based on comments from our second reviewer, we removed odds ratios from this paper and instead report unadjusted differences in the characteristics of patients who tested positive versus negative (now described in line 191).

**8. In discussion: I would like you to remark that the mode of delivery and preterm delivery were not related to SARS-COV-2 positivity (findings contrary to the descriptions in China and other places outside USA). Was the length of stay greater in the symptomatic patients due to the severity of illness? Was the criteria for discharge different in this population?**

The length of stay was greater for symptomatic patients:

LOS among symptomatic patients: mean = 3.92, std dev = 2.10; median = 4, IQR = 2-6

LOS among asymptomatic patients: mean = 2.72, std dev = 1.96; median = 2, IQR = 2-3

The increased length of stay in the symptomatic patients is likely secondary to the severity of illness being greater in symptomatic patients. In order to warrant admission for SARS-Cov-2 symptoms typically maternal fever had to be accompanied by either maternal or fetal tachycardia, shortness of breath or decreased oxygen saturation on room air, or concerning findings on chest X-ray for possible pneumonia. Patients who did not meet these criteria, and had normal vital signs apart from fever, and reassuring fetal testing were typically assessed in our triage area and discharged home rather than admitted. These patients who were not admitted would not have been included in this cohort. The criteria for discharge among symptomatic patients included normalization of vital signs, specifically remaining afebrile and having oxygen saturation of over 95% consistently on room air both at rest and with ambulation.

We now remark on this in the discussion section as requested (line 334).

**9. Line 204: Discussion: Which kind of facemasks? (surgical, isolation, dental, etc).**

Infected and PUI mothers were provided with surgical facemasks. This was clarified in the discussion text (line 383).

**10. Figure 2: In S ARS-CoV-2 positive line, in the row counseling, mother wears which kind of mask?**

Surgical mask. This was clarified in the figure.

**In line SARS-CoV-2 negative row counseling, you counseled normal postpartum vaccination?**

Yes, for the women who tested negative routine postpartum vaccination counseling and administration (if indicated) was performed. This was clarified (line 382).

**In line Person Under Investigation, row Health worker PPE, in the other interaction, which kind of mask was used? The same for the mother.**

Health workers used N95 masks for any PUI once available, and mothers used surgical masks.

This was clarified in the figure.

**11. Minor Observations: language and readibility lines 58-62, line 103, line 109, line 190, line 203, Table 2: line SARS-CoV-2 positive row Counseling, line PUI row Counseling and Follow-up, line 226.**

We edited the text as requested for all lines to improve the readability: Lines 58-62, Line 103, Line 109, Line 190, Line 203, and Line 226.

Table 2 line SARS-CoV-2 positive counseling: we edited this to improve readability.

Table 2 line PUI row counseling and follow up: we edited this to improve readability.

**12. Minor Observations: please correct references 9, 11-13.**

References 9, 11, and 12 were corrected. We note there is not a reference 13.

**Reviewer #2: I have read the article Universal screening for SARS-CoV-2 infection among pregnant women at Elmhurst Hospital Center, Queens, New York.
This is a cross-sectional study evaluating the universal screening of SARS-CoV-2 among obstetric population attending a reference center from NY.
The article is well written, the topic is adequate for the situation we are going through and it is highly relevant.

1. Although, the study is a cross-sectional, it seems in the analysis to be an analytical cross-sectional. The authors use two main outcomes, the first is the positive SARS-CoV-2 infection, and the second the presence of symptoms. It does not seem right. If this is an analytical cross-sectional, all population is supposed to be screened at arrival in which exposure and outcome are measured at the same time. As judged by your diagram, it seems that the exposures were symptomatic and asymptomatic pregnant, in which the main outcome would be the positive or negative test.**

Thank you for this constructive and helpful feedback. We have changed the analysis to be consistent with a descriptive cross-sectional study throughout the manuscript. In particular, we no longer assess odds-ratios and instead compare the characteristics of patients who tested positive versus negative and report unadjusted differences, with 95% CIs from a 2 sided, unpaid t-test (now described in line 175).

**About reporting:

2. Title and abstracts are adequate. Mention of the study design as well as informative and balanced summary are present. Although, the study design is not adequate.**

As stated above, we have changed the analysis to match the study design of a descriptive cross-sectional study.

**3. Background/rationale is adequate. Nevertheless, the objective only describes the population and outcome of interest (it seems that the original idea of the authors was to assess only population and outcome as a descriptive cross-sectional and not an analytical in which exposures are also measures). In this sense. If the authors intended to perform only a descriptive cross-sectional, all statistical inference in beyond the capability of the design and the odds ratios should be taken out. Statistical inferences like odds ratio, are meant only for analytical studies in which there are two groups of exposures (exposed and controls).**

We have removed the odds ratios.

**4. Study design: The PECOS is compatible with an analytical cross-sectional study as judged by the analysis and results, but not by the objective.**

**Population: All pregnant women attending the hospital
Exposure: symptomatic
Controls: asymptomatic
Outcome: positive rt-PCR for SARS-CoV-2
Participants: Study design and setting have repetitive information with participants and data collection.**
**Variables: Please, clearly define all outcomes, exposures, potential confounders. Describe all variables and definitions.**

**Data sources: adequate.
Study size: please describe the study size or if not calculated, reasons for not doing it.**

We now refer to the study design as descriptive cross-sectional.

We have clarified the definition of the two outcome variables (line 148): (1) SARS-CoV-2 positivity, stratified by demographic characteristics, maternal comorbidities, and delivery outcomes; and (2) Symptomatic or asymptomatic presentation at the time of testing among SARS-CoV-2 positive women. We deleted the repetitive information from the “Study design and setting” section. There is not an exposure or control variable in this study.

We included all pregnant women (N=126) admitted to the L&D Unit at Elmhurst Hospital from March 29, 2020 to April 22, 2020. This time period was selected as it comprised the period of universal screening during the ‘surge’ of SARS-Cov-2 cases in NYC. Study size was limited by the time period.

We clarified this in the text (line 141).

**5. Biases: please describe potential biases that addresses selection bias, recall bias, and so.**

Selection bias may be present as pregnant women with SARS-CoV-2 infection or symptoms may have been more or less likely to present for admission. We have added this to the discussion (line 300).

**6. Statistical methods: If this is an analytical cross-sectional, odds ratios are adequate. If this is a descriptive cross-sectional, odds ratios should be taken out.**

Odds ratios were removed.

**7. About results:**

**Participants are described. All the initial description as well as the first table are compatible with Population and outcome, adequately describing the data. In this case, odds ratio is inadequate.**

**After table 1, data are now divided by population, exposed to symptoms or no symptoms, and those deriving in positive or negative PCR as the main outcome.

Define the study design adequately first and then proceed to the analysis.**

**The rest of the results are adequate.**

We removed the odds ratios and have revised the analysis to be consistent with a descriptive cross-sectional design.

**8. Discussion. Please, add strengths and limitations. Please discuss as a limitation the use of rapid test for SARS-CoV-2 compared to non-rapid tests.**

We now describe strengths and limitations of the study into the Discussion, including the point about using a rapid test (line 357).

**9. Please describe the possible reasons for the higher prevalence of positive tests among the Spanish speaking community.**

We have added this to the discussion (line 328).

**10. Figure 2 and all its description is beyond the objective and scope of the manuscript. Please remove figure 2 and the surrounding discussion.**

We value your feedback on this, however we prefer to leave this information in the manuscript as we believe it is critically important to the manuscript and highly relevant to clinical readers of *PLOS One*. We fully defer to the Editors on the final decision, but note that many practitioners are facing high rates of SARS-CoV-2 infection in their hospitals now and others will be in the near future, and there is an important connection between the data on universal screening and the implications for a clinical response. Given that our other reviewer did not ask us to remove this section, and that we believe it is critically important to the paper, we prefer to keep it in if feasible.

**11. Discussion seems adequate, the problem is that the discussion is compatible with a descriptive cross-sectional study in which only population and outcome are assessed at the same time. This differs from the results as they seem to be derived from an analytical study.**

We have revised the analysis to be consistent with a descriptive cross-sectional study.

**Please add any findings

General comments:

12. Please use prevalence instead of incidence. Incidence implies a time period and this is not quite compatible with a cross-sectional study. It would be better to say prevalence.**

We have replaced incidence with prevalence in the manuscript (line 118). **13. The study design differs in the main objective, methods, the way results are presented, and conclusion. Please, choose one design and analyze the data according to it.**

We have revised the manuscript to have one study design: descriptive cross-sectional design.
