# Supplementary material for: Universal screening for SARS-CoV-2 infection among pregnant women at Elmhurst Hospital Center, Queens, New York
Source: PLoS One. 2020 Dec 10;15(12):e0238409. doi: 10.1371/journal.pone.0238409 (PMC7728244; doi:10.1371/journal.pone.0238409)
Supplement: Peer review history

# Peer review: Decision Letter 0

**DOI:** 10.1371/journal.pone.0238409.r001

**Authors:** Rogelio Cruz-Martinez

**Transfer Alert**

This paper was transferred from another journal. As a result, its full editorial history (including decision letters, peer reviews and author responses) may not be present.

16 Jun 2020

PONE-D-20-14679

Universal screening for SARS-CoV-2 infection among pregnant women at Elmhurst Hospital Center, Queens, New York

PLOS ONE

Dear Dr. Maru,

Thank you for submitting your manuscript to PLOS ONE. After careful consideration, we feel that it has merit but does not fully meet PLOS ONE’s publication criteria as it currently stands. Therefore, we invite you to submit a revised version of the manuscript that addresses the points raised during the review process.

This very-well written manuscript add interesting information regarding the clinical relevance of universal screening for SARS2-Covid infection in pregnant women. The authors aimed to assess the incidence of SARS2-Covid infection according to the socio-demographic data. I believe that this information is relevant for clinicians attending pregnant women in this pandemic era and thus, the manuscript could be suitable for publication in this Journal after including all the reviewer´s corrections.

Please submit your revised manuscript by Jul 31 2020 11:59PM. If you will need more time than this to complete your revisions, please reply to this message or contact the journal office at plosone@plos.org. When you're ready to submit your revision, log on to https://www.editorialmanager.com/pone/ and select the 'Submissions Needing Revision' folder to locate your manuscript file.

Please include the following items when submitting your revised manuscript:

- A rebuttal letter that responds to each point raised by the academic editor and reviewer(s). You should upload this letter as a separate file labeled 'Response to Reviewers'.
- A marked-up copy of your manuscript that highlights changes made to the original version. You should upload this as a separate file labeled 'Revised Manuscript with Track Changes'.
- An unmarked version of your revised paper without tracked changes. You should upload this as a separate file labeled 'Manuscript'.

If you would like to make changes to your financial disclosure, please include your updated statement in your cover letter. Guidelines for resubmitting your figure files are available below the reviewer comments at the end of this letter.

If applicable, we recommend that you deposit your laboratory protocols in protocols.io to enhance the reproducibility of your results. Protocols.io assigns your protocol its own identifier (DOI) so that it can be cited independently in the future. For instructions see: http://journals.plos.org/plosone/s/submission-guidelines#loc-laboratory-protocols

We look forward to receiving your revised manuscript.

Kind regards,

Rogelio Cruz-Martinez, Ph.D.

Academic Editor

PLOS ONE

Journal Requirements:

When submitting your revision, we need you to address these additional requirements.

1. Please ensure that your manuscript meets PLOS ONE's style requirements, including those for file naming. The PLOS ONE style templates can be found at https://journals.plos.org/plosone/s/file?id=wjVg/PLOSOne_formatting_sample_main_body.pdf and https://journals.plos.org/plosone/s/file?id=ba62/PLOSOne_formatting_sample_title_authors_affiliations.pdf

2. We note that you have indicated that data from this study are available upon request. PLOS only allows data to be available upon request if there are legal or ethical restrictions on sharing data publicly. For information on unacceptable data access restrictions, please see http://journals.plos.org/plosone/s/data-availability#loc-unacceptable-data-access-restrictions.

In your revised cover letter, please address the following prompts:

a) If there are ethical or legal restrictions on sharing a de-identified data set, please explain them in detail (e.g., data contain potentially identifying or sensitive patient information) and who has imposed them (e.g., an ethics committee). Please also provide contact information for a data access committee, ethics committee, or other institutional body to which data requests may be sent.

b) If there are no restrictions, please upload the minimal anonymized data set necessary to replicate your study findings as either Supporting Information files or to a stable, public repository and provide us with the relevant URLs, DOIs, or accession numbers. Please see http://www.bmj.com/content/340/bmj.c181.long for guidelines on how to de-identify and prepare clinical data for publication. For a list of acceptable repositories, please see http://journals.plos.org/plosone/s/data-availability#loc-recommended-repositories.

We will update your Data Availability statement on your behalf to reflect the information you provide.

Additional Editor Comments (if provided):

I strongly suggest including the socio-economic status and if possible the number of persons per households of the studied population in order to evaluate if hyspanic ethnicity and Spanish as a primary language are or not confounder associated factors. 

[Note: HTML markup is below. Please do not edit.]

Reviewers' comments:

Reviewer's Responses to Questions

**Comments to the Author**

1. Is the manuscript technically sound, and do the data support the conclusions?

The manuscript must describe a technically sound piece of scientific research with data that supports the conclusions. Experiments must have been conducted rigorously, with appropriate controls, replication, and sample sizes. The conclusions must be drawn appropriately based on the data presented. 

Reviewer #1: Yes

Reviewer #2: Partly

**********

2. Has the statistical analysis been performed appropriately and rigorously? 

Reviewer #1: Yes

Reviewer #2: No

**********

3. Have the authors made all data underlying the findings in their manuscript fully available?

The PLOS Data policy requires authors to make all data underlying the findings described in their manuscript fully available without restriction, with rare exception (please refer to the Data Availability Statement in the manuscript PDF file). The data should be provided as part of the manuscript or its supporting information, or deposited to a public repository. For example, in addition to summary statistics, the data points behind means, medians and variance measures should be available. If there are restrictions on publicly sharing data—e.g. participant privacy or use of data from a third party—those must be specified.

Reviewer #1: Yes

Reviewer #2: No

**********

4. Is the manuscript presented in an intelligible fashion and written in standard English?

PLOS ONE does not copyedit accepted manuscripts, so the language in submitted articles must be clear, correct, and unambiguous. Any typographical or grammatical errors should be corrected at revision, so please note any specific errors here.

Reviewer #1: Yes

Reviewer #2: Yes

**********

5. Review Comments to the Author

Please use the space provided to explain your answers to the questions above. You may also include additional comments for the author, including concerns about dual publication, research ethics, or publication ethics. (Please upload your review as an attachment if it exceeds 20,000 characters)

Reviewer #1: Comments to the Author:

The study by Dr. Maru et al. describes the prevalence of SARS-COV-2 infection among pregnant patients that were admitted to a L&D room, at the epicenter of the COVID-19 pandemia in USA. The authors add very valuable information regarding the spectrum of COVID-19 disease in this population; they also address demographic characteristics to their findings.

I have some suggestions and questions:

1. Line 91: past tense. “The primary objective of this study was to describe”.

2. Line 111: In Methods: could you describe were the swabs were taken (nasopharyngeal, oropharyngeal, etc)?

3. Line 116: Comorbidities: could you describe which comorbidities were assessed?

4. Line 133: Table 1. Title: Were all the patients presenting for delivery? You report 12 still pregnant patients. Table 1 per se: Comorbidities (No.) means the number of comorbidities, then you report 1+, this means all the patients had 2 or more comorbidities or had at least one? Could you be more clear? If you wish to express it as yes / none, in the results text you may describe the comorbidities and the presence of more than 1, and may be usefull in this scenario to eliminate the Word (No.) in the table.

5. Line 140: Results: Concerning the symptomatic patients, could you be more thorough about the symptoms? Was the presence of symptoms related to the mode of delivery/preterm delivery? Any fatalities?

6. Line 148: Results: I don´t understand the variable primary language (Spanish) and its relationship with positivity testing: does this mean these patients were not born in USA or are first degree descedants of latin american inmigrants, and this relates these characteristics with positivity? Is there a way you could you be more elaborate?

7. Lines 149-151: Results: could you add the ORs to the description?

8. In discussion: I would like you to remark that the mode of delivery and preterm delivery were not related to SARS-COV-2 positivity (findings contrary to the descriptions in China and other places outside USA). Was the length of stay greater in the symptomatic patients due to the severity of illness? Was the criteria for discharge different in this population?

9. Line 204: Discussion: Which kind of facemasks? (surgical, isolation, dental, etc).

10. Figure 2: In SARS-CoV-2 positive line, in the row counseling, mother wears which kind of mask?

In line SARS-CoV-2 negative row counseling, you counseled normal postpartum vaccination?

In line Person Under Investigation, row Health worker PPE, in the other interaction, which kind of mask was used? The same for the mother.

11. Minor Observations: language and readibility lines 58-62, line 103, line 109, line 190, line 203, Table 2: line SARS-CoV-2 positive row Counseling, line PUI row Counseling and Follow-up, line 226.

12. Minor Observations: please correct references 9, 11-13.

Reviewer #2: I have read the article Universal screening for SARS-CoV-2 infection among pregnant women at Elmhurst Hospital Center, Queens, New York.

This is a cross-sectional study evaluating the universal screening of SARS-CoV-2 among obstetric population attending a reference center from NY.

The article is well writting, the topic is adequate for the situation we are going through and it is highly relevant.

Although, the study is a cross-sectional, it seems in the analysis to be an analytical cross-sectional. The authors use two main outcomes, the first is the positive SARS-CoV-2 infection, and the second the presence of symptoms. It does not seem right. If this is an analytical cross-sectional, all population is supposed to be screened at arrival in which exposure and outcome are measured at the same time. As judged by your diagram, it seems that the exposures were symptomatic and asymptomatic pregnant, in which the main outcome would be the positive or negative test.

About reporting:

Title and abstracts are adequate. Mention of the study design as well as informative and balanced summary are present. Although, the study design is not adequate.

Background/rationale is adequate. Nevertheless, the objective only describes the population and outcome of interest (it seems that the original idea of the authors was to assess only population and outcome as a descriptive cross-sectional and not an analytical in which exposures are also measures). In this sense. If the authors intended to perform only a descriptive cross-sectional, all statistical inference in beyond the capability of the design and the odds ratios should be taken out. Statistical inferences like odds ratio, are meant only for analytical studies in which there are two groups of exposures (exposed and controls).

Study design: The PECOS is compatible with an analytical cross-sectional study as judged by the analysis and results, but not by the objective.

Population: All pregnant women attending the hospital

Exposure: symptomatic

Controls: asymptomatic

Outcome: positive rt-PCR for SARS-CoV-2

Participants: Study design and setting have repetitive information with participants and data collection.

Variables: Please, clearly define all outcomes, exposures, potential confounders. Describe all variables and definitions.

Data sources: adequate.

Study size: please describe the study size or if not calculated, reasons for not doing it.

Biases: please describe potential biases that addresses selection bias, recall bias, and so.

Statistical methods: If this is an analytical cross-sectional, odds ratios are adequate. If this is a descriptive cross-sectional, odds ratios should be taken out.

About results:

Participants are described. All the initial description as well as the first table are compatible with Population and outcome, adequately describing the data. In this case, odds ratio is inadequate. After table 1, data are now divided by population, exposed to symptoms or no symptoms, and those deriving in positive or negative PCR as the main outcome.

Define the study design adequately first and then proceed to the analysis.

The rest of the results are adequate.

Discussion. Please, add strengths and limitations. Please discuss as a limitation the use of rapid test for SARS-CoV-2 compared to non-rapid tests.

Please describe the possible reasons for the higher prevalence of positive tests among the Spanish speaking community.

Figure 2 and all its description is beyond the objective and scope of the manuscript. Please remove figure 2 and the surrounding discussion.

Discussion seems adequate, the problem is that the discussion is compatible with a descriptive cross-sectional study in which only population and outcome are assessed at the same time. This differs from the results as they seem to be derived from an analytical study.

Please add any findings

General comments:

Please use prevalence instead of incidence. Incidence implies a time period and this is not quite compatible with a cross-sectional study. It would be better to say prevalence.

The study design differs in the main objective, methods, the way results are presented, and conclusion. Please, choose one design and analyze the data according to it.

**********

6. PLOS authors have the option to publish the peer review history of their article (what does this mean?). If published, this will include your full peer review and any attached files.

If you choose “no”, your identity will remain anonymous but your review may still be made public.

**Do you want your identity to be public for this peer review?** For information about this choice, including consent withdrawal, please see our Privacy Policy.

Reviewer #1: Yes: Rosa Villalobos-Gómez

Reviewer #2: No

[NOTE: If reviewer comments were submitted as an attachment file, they will be attached to this email and accessible via the submission site. Please log into your account, locate the manuscript record, and check for the action link "View Attachments". If this link does not appear, there are no attachment files.]

While revising your submission, please upload your figure files to the Preflight Analysis and Conversion Engine (PACE) digital diagnostic tool, https://pacev2.apexcovantage.com/. PACE helps ensure that figures meet PLOS requirements. To use PACE, you must first register as a user. Registration is free. Then, login and navigate to the UPLOAD tab, where you will find detailed instructions on how to use the tool. If you encounter any issues or have any questions when using PACE, please email PLOS at figures@plos.org. Please note that Supporting Information files do not need this step.

# Author comment: Author response to Decision Letter 0

**DOI:** 10.1371/journal.pone.0238409.r002

31 Jul 2020

I have attached a document "Response to Reviewers" with all responses.

Attachment Submitted filename: Response to Reviewers 7.27.20.docx Click here for additional data file.

# Editor report: Decision Letter 1

**DOI:** 10.1371/journal.pone.0238409.r003

**Authors:** Rogelio Cruz-Martinez

18 Aug 2020

Universal screening for SARS-CoV-2 infection among pregnant women at Elmhurst Hospital Center, Queens, New York

PONE-D-20-14679R1

Dear Dr. Maru,

We’re pleased to inform you that your manuscript has been judged scientifically suitable for publication and will be formally accepted for publication once it meets all outstanding technical requirements.

Within one week, you’ll receive an e-mail detailing the required amendments. When these have been addressed, you’ll receive a formal acceptance letter and your manuscript will be scheduled for publication.

An invoice for payment will follow shortly after the formal acceptance. To ensure an efficient process, please log into Editorial Manager at http://www.editorialmanager.com/pone/, click the 'Update My Information' link at the top of the page, and double check that your user information is up-to-date. If you have any billing related questions, please contact our Author Billing department directly at authorbilling@plos.org.

If your institution or institutions have a press office, please notify them about your upcoming paper to help maximize its impact. If they’ll be preparing press materials, please inform our press team as soon as possible -- no later than 48 hours after receiving the formal acceptance. Your manuscript will remain under strict press embargo until 2 pm Eastern Time on the date of publication. For more information, please contact onepress@plos.org.

Kind regards,

Rogelio Cruz-Martinez, Ph.D.

Academic Editor

PLOS ONE

# Editor report: Acceptance letter

**DOI:** 10.1371/journal.pone.0238409.r004

**Authors:** Rogelio Cruz-Martinez

3 Dec 2020

PONE-D-20-14679R1 

Universal screening for SARS-CoV-2 infection among pregnant women at Elmhurst Hospital Center, Queens, New York 

Dear Dr. Maru:

I'm pleased to inform you that your manuscript has been deemed suitable for publication in PLOS ONE. Congratulations! Your manuscript is now with our production department. 

If your institution or institutions have a press office, please let them know about your upcoming paper now to help maximize its impact. If they'll be preparing press materials, please inform our press team within the next 48 hours. Your manuscript will remain under strict press embargo until 2 pm Eastern Time on the date of publication. For more information please contact onepress@plos.org.

If we can help with anything else, please email us at plosone@plos.org. 

Thank you for submitting your work to PLOS ONE and supporting open access. 

Kind regards, 

PLOS ONE Editorial Office Staff

on behalf of

Dr Rogelio Cruz-Martinez 

Academic Editor

PLOS ONE
